# Supplementary material for: 4D Printing of Electroactive Triple-Shape Composites
Source: Polymers (Basel). 2023 Feb 7;15(4):832. doi: 10.3390/polym15040832 (PMC9961650; doi:10.3390/polym15040832)
Supplement: Supplementary file 1 [file polymers-15-00832-s001.zip › polymers-2197443-supplementary.pdf]

Supplementary Materials: 4D Printing of Electroactive Triple-Shape Composites

Muhammad Yasar Razzaq [1*], Joamin Gonzalez-Gutierrez[1*], Muhammad Farhan[2*], Rohan Das[1,3], David Ruch[1], Stephan Westermann [1] and Daniel F. Schmidt [1*]

[1] Department of Materials Research and Technology, Luxembourg Institute of Science and Technology, ZAE Robert Steichen, L-4940 Hautcharage, Luxembourg
[2] Institute of Active Polymers, Helmholtz-Zentrum Hereon, D-14513 Teltow, Germany
[3] Department of Physics and Materials Science, University of Luxembourg, L-4365 Esch-sur-Alzette, Luxembourg
* Correspondence: yasar.razzaq@list.lu (M.Y.R.); daniel.schmidt@list.lu (D.F.S.)

Table S1. Printing parameters used for red coloured PLA and the conductive composites.

Parameter	Red coloured PLA	Conductive composite
Layer height (mm) - all	0.25	0.25
Line width (mm) - all	0.4	0.8
Wall thickness (mm)	0.4	0.8
Wall line count	2	2
Top surface skin layers	2	2
Bottom layer	2	2
Top/Bottom Pattern	Lines	Lines
Infill density (%)	50	100
Infill pattern	Gyrod	Lines
Printing temperature (°C) - all	210	245
Flow (%) - all	100	100
Print speed (mm/s)	30	15
Outer/Inner Wall speed (mm/s)	25	12
Initial layer speed (mm/s)	25	7.5
Skirt/Brim speed (mm/s)	25	25
Retraction distance (mm)	1.4	6.5
Retraction speed (mm/s)	25	25
Fan speed (%)	100	100
Build plate adhesion type	Skirt	Skirt
Skirt line count	1	1
Skirt distance (mm)	3	3
Nozzle switch retraction distance (mm)	16	16
Nozzle switch retraction speed (mm/s)	20	20

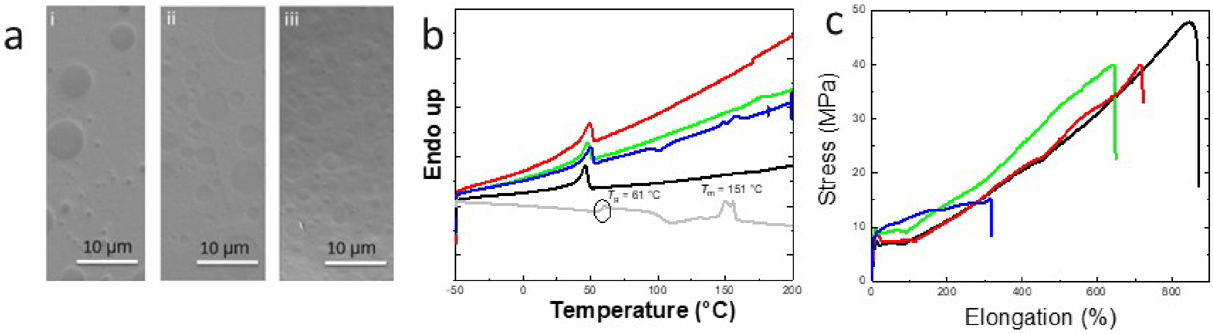

Figure S1. a) Back-scatter SEM images of the unfilled pure blends, i) PEU90PLA10, ii) PEU80PLA20, iii) PEU70PLA30, b) DSC thermograms of neat PEU (black line), neat PLA (grey line), PEU90PLA10 (red line), PEU80PLA20(green line), PEU70PLA30 Blue line) (c) Tensile testing of the pure blends pure PEU (black line) PEU90PLA10 (red line), PEU80PLA20(green line), PEU70PLA30 (blue line).

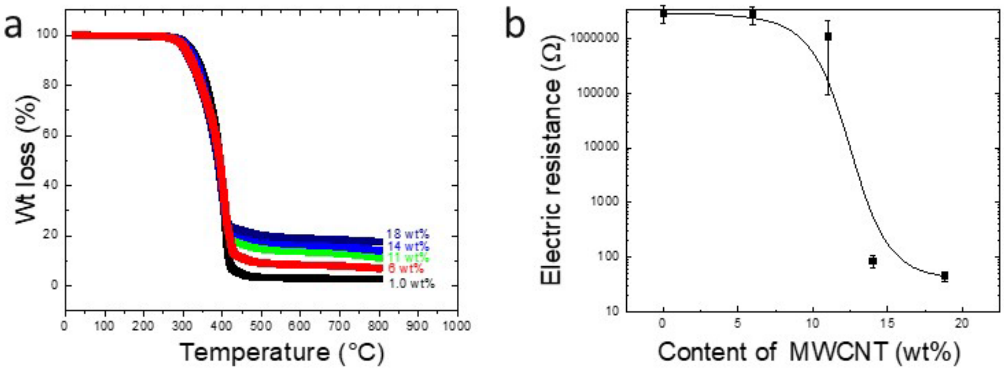

Figure S2. a) Thermogravimetric analysis (TGA) of the pure blend PEU70PLA30 (Black line) and composite PEU70PLA30MWCNT6 (red line), PEU70PLA30MWCNT11 (green line), PEU70PLA30MWCNT14 (blue line), PEU70PLA30MWCNT18 (navy line), b) Dependence of electric resistance at room temperature of PEU70PLA30 and MWCNT composites. Symbols represent measurement values; the line is a spline curve.

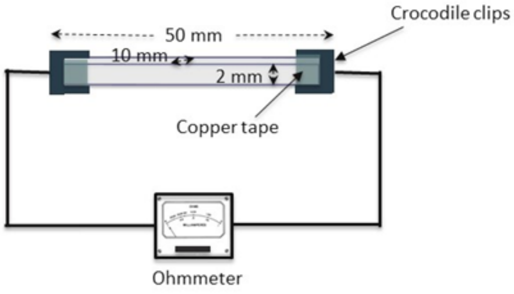

Figure S3. Schematic representation of the method used for measuring the resistance of the composite sample.

S1. Morphology of the reference samples by atomic force microscopy

PEUs possess a microphase-separated structure consisting of a soft segment matrix and hard segment domains [55–57]. For the neat PEU, phase contrast can be detected in Figure S4d, with the hard segments appearing as the minority phase (dark brown) and the soft segments appearing as the majority phase (light brown). The topographic images of neat blend PEU70PLA30 (Figure S4b) revealed the presence of PLA as spherical domains in the PEU matrix, indicating an immiscible blend, as was also seen via SEM analysis. In addition, the phase contrast images (Figure S4e) revealed the hard segments of the PEU, which were observed to give similar phase contrast to the PLA domains, given their

greater rigidity in comparison with the elastomeric nature of the PEU soft segments that make up the majority of the (brighter) matrix [58].

To better understand the dispersion and distribution of the MWCNTs in the PEU matrix, a composite of pure PEU and MWCNTs (PEU100MWCNT14) was analyzed by AFM. Figure S4c represents topographic image of the PEU100MWCNT14 specimen, where interlaced cylindrical structures corresponding to the MWCNTs are visible [60]. Compared to the neat blend PEU70PLA30 (Figure S4b), the presence of MWCNTs is prominent in the PEU100MWCNT14 blend, where the fillers display a higher height profile in Figure S4c. Also, the magnified phase contrast image (Fig. S4f) clearly shows the rigid MWCNTs, which appear dark compared to the matrix [59,60]. Their high concentration and close proximity are consistent with the formation of the percolated filler network that enables Joule heating. However, compared to image S4e, the PEU hard segment could not be seen in Figure S4f.

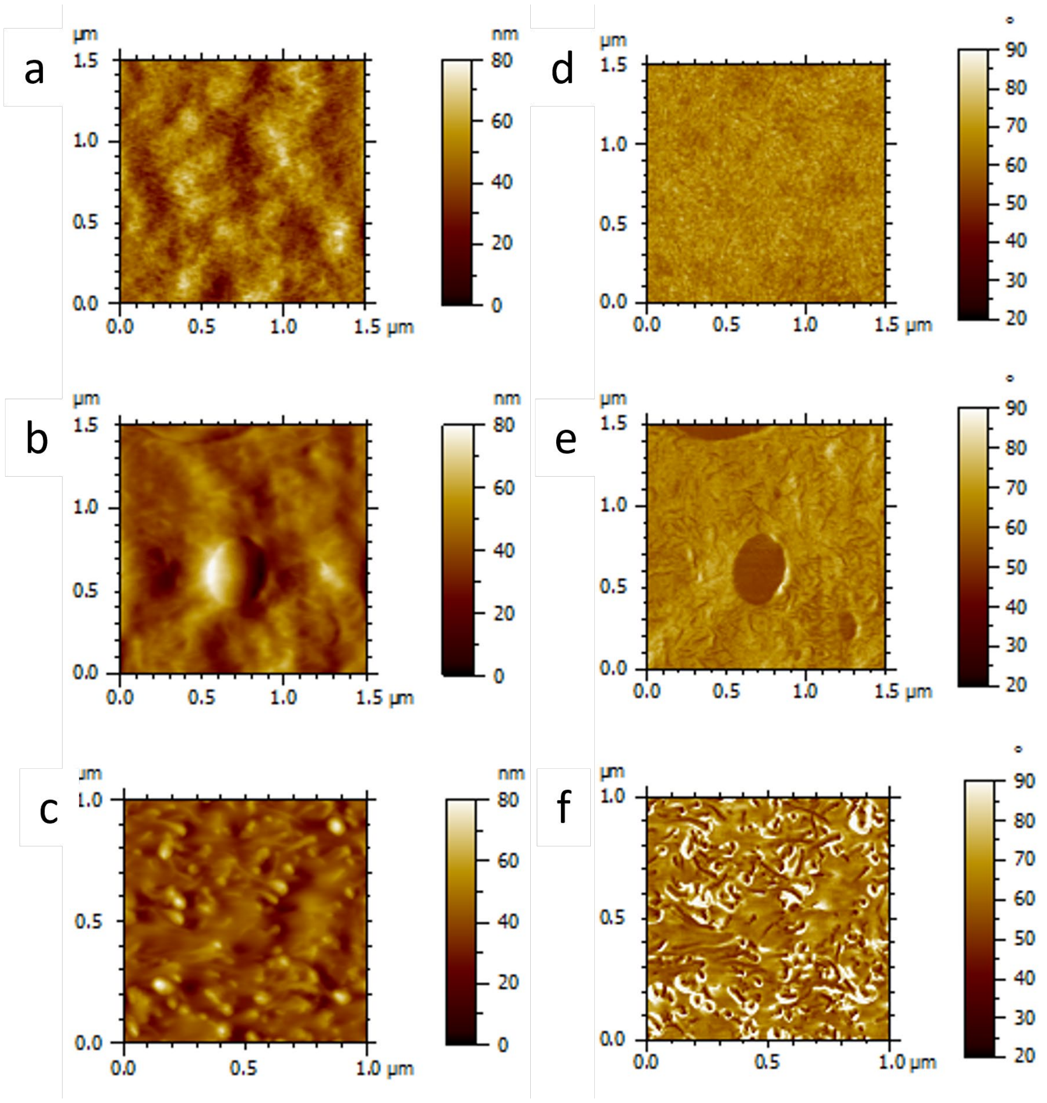

Figure S4. AFM images of the neat PEU (a,d); neat PEU70PLA30 blend (b, e); composite PEU100MWCNT14 (c, f). Images a - c are topography images and (d-f) are phase contrast images.

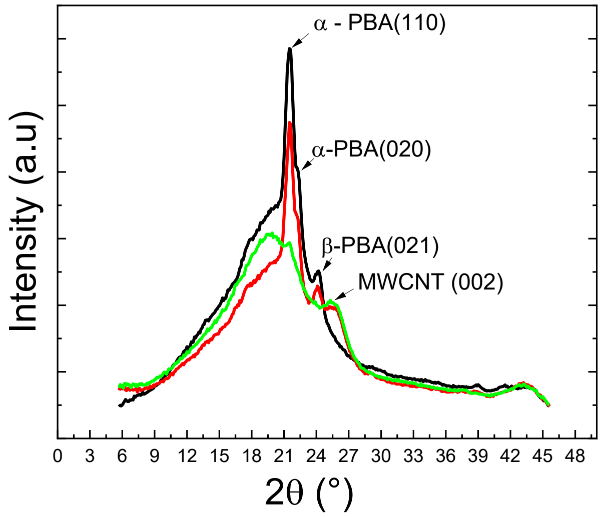

Figure S5. WAXS analysis of neat blend PEU70PLA30 at ambient temperature (black line) and composite PEU70PLA30MWCNT14 at ambient temperature (red line) and at 55 °C (green line).

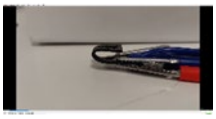

Video S1: Electrically activated triple-shape effect (TSE) in 3D printed "U" shape model.

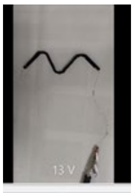

Video S2: Electric activation of the TSE in 3D printed "M" shaped compression device.

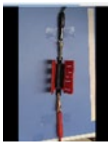

Video S3: Electric activation of the TSE in a multi-material hinge structure.
